# Establishing a Prognostic Model Based on Ulceration and Immune Related Genes in Melanoma Patients and Identification of EIF3B as a Therapeutic Target

**DOI:** 10.3389/fimmu.2022.824946

**Published:** 2022-02-22

**Authors:** Zhengquan Wu, Ke Lei, Sheng Xu, Jiali He, Enxian Shi

**Affiliations:** ^1^ Walter Brendel Center for Experimental Medicine, University of Munich, Munich, Germany; ^2^ Department of Otorhinolaryngology, Head and Neck Surgery, University of Munich, Munich, Germany; ^3^ Department of Dermatology, The Second People’s Hospital of Chengdu, Chengdu, China; ^4^ Patient Monitor and Life Supporting (PMLS), Shenzhen Mindray Bio-Medical Electronics Co., Ltd, Shenzhen, China; ^5^ Department of General Outpatient, Shen zhen Healthcare Committee Office, Shenzhen, China

**Keywords:** ulceration, melanoma, immunity, immunotherapy, EIF3B, prognostic model

## Abstract

Ulceration and immune status are independent prognostic factors for survival in melanoma patients. Herein univariate Cox regression analysis revealed 53 ulcer-immunity-related DEGs. We performed consensus clustering to divide The Cancer Genome Atlas (TCGA) cohort (n = 467) into three subtypes with different prognosis and biological functions, followed by validation in three merged Gene Expression Omnibus (GEO) cohorts (n = 399). Multiomics approach was used to assess differences among the subtypes. Cluster 3 showed relatively lesser amplification and expression of immune checkpoint genes. Moreover, Cluster 3 lacked immune-related pathways and immune cell infiltration, and had higher proportion of non-responders to immunotherapy. We also constructed a prognostic model based on ulceration and immune related genes in melanoma. EIF3B was a hub gene in the intersection between genes specific to Cluster 3 and those pivotal for melanoma growth (DepMap, https://depmap.org/portal/download/). High EIF3B expression in TCGA and GEO datasets was related to worst prognosis. *In vitro* models revealed that EIF3B knockdown inhibited melanoma cell migration and invasion, and decreased TGF-β1 level in supernatant compared with si-NC cells. EIF3B expression was negatively correlated with immune-related signaling pathways, immune cell gene signatures, and immune checkpoint gene expression. Moreover, its low expression could predict partial response to anti-PD-1 immunotherapy. To summarize, we established a prognostic model for melanoma and identified the role of EIF3B in melanoma progression and immunotherapy resistance development.

## Introduction

Melanoma is a highly aggressive skin malignant tumor that is prone to metastasis at an early stage and has the highest mortality rate among skin cancers (1). In recent years, its incidence has increased, and it has become one of the fastest growing tumors (2). The rate of diagnosis among adolescents is also increasing (3). The prognostic risk factors of malignant melanoma include fraction of tumor infiltrating lymphocytes (4, 5), and tumor stage including tumor thickness and ulceration (6). Although immunotherapy (e.g., anti-PD-1 therapy) has been found to significantly reduce the mortality of melanoma in recent years (7), numerous patients still develop resistance, and thus, the mortality rate continues to remain high (8).

It has been shown that ulceration is correlated to worse overall survival in melanoma, and it plays as an independent prognostic factor of melanoma (9, 10). Moreover, according to previous studies, immune cell infiltration in patients with melanoma is related to an improvement in the survival rate and response to immunotherapy (4, 11). More interestingly, ulceration has been proven to help build an immunosuppressive microenvironment for melanoma. Ulcerated melanoma is associated with higher infiltration of immunosuppressive cells (12), such as tumor-associated macrophages (13) and Treg cells, but lesser infiltration of GZMB+ and cytotoxic CD8+ T cells (14). The low tumor infiltrating lymphocytes combined with presence of ulceration evidently accelerates melanoma progression (4, 15). Therefore, it is necessary to comprehensively understand the molecular characteristics of patients showing a combination of ulceration and immune cell infiltration.

To provide important insights into the molecular characteristics of ulcers and immune cell infiltration in patients, we comprehensively identified three different Clusters (Cluster1, Cluster2, Cluster3) based on ulcer-immunity-related DEGs. Then we analyzed differences in prognosis, genomic profiles, immune cell infiltration, and immunotherapy response among the three subtypes. EIF3B was then identified as the hub gene of the subtype related with the worst prognosis in melanoma, which provided a clue that EIF3B could be a potential therapeutic drug target in melanoma. EIF3B, a subunit of the eIF3 translation initiation factor complex, is particularly essential as it serves a critical scaffolding function for the entire eIF3 complex (16). EIF3B is evidently overexpressed in various human cancers and acts as an oncogene to promote their invasion and metastasis. Wang et al. proved that EIF3B is upregulated in prostate and bladder cancer tissues and that it promotes bladder and prostate cancer growth and lung metastasis/colonization by regulating the expression of integrin α5 and cell cycle-related proteins (17). Besides, Ma et al. proved that EIF3B regulates various cancer-related pathways to promote gastric cancer occurrence and development (18); However, its role in melanoma remains unclear.

This study revealed that EIF3B expression was negatively correlated with immune-related signaling pathways, immune cell gene signatures, and immune checkpoint gene expression. EIF3B knockdown also inhibited the migration and invasion of melanoma cells *in vitro*, and decreased concentration of TGF-β1 in supernatant. And we further constructed and verified a prognostic model based on the ulceration and immune related genes in melanoma. Our prognostic model could provide meaningful prognostic value in clinical application, and our findings also highlight the potential role of EIF3B in melanoma progression.

## Materials and Methods

### Dataset Source and Preprocessing

Overall, 937 patients from six suitable skin cutaneous melanoma (SKCM) cohorts [GSE65904, GSE59455, GSE19234, GSE78220, GSE91061, GSE54467, and The Cancer Genome Atlas (TCGA)-SKCM] were subjected to analyses. Patients without survival information and RNA-seq data were excluded. For the dataset from Gene Expression Omnibus (GEO), we downloaded preprocessed clinical and transcriptome data using the R ‘GEOquery’ package (19) and merged the related GEO dataset using the ‘ComBat’ algorithm from the R ‘sva’ package (20). As for the dataset from TCGA, we used the R ‘TCGAbiolinks’ package (21) to download all available transcriptome [fragments per kilobase of transcript per million fragments mapped (FPKM) value] and clinical data from the Genomic Data Commons (GDC, https://portal.gdc.cancer.gov/). Furthermore, clinical information pertaining to ulceration in TCGA patients was extracted from the UCSC Cancer Browser (XENA, https://xenabrowser.net/datapages/). FPKM values were then converted to transcripts per million (TPM) values, which were used in subsequent analyses.

### Identification of Differentially Expressed Genes (DEGs)

We used the R ‘TCGAbiolinks’ package (21) to download raw read counts for TCGA-SKCM patients from the GDC. The R ‘DESeq2’ package (22) was applied to standardize the read counts and perform differential gene analysis. And for the multiclass DEseq2, we only took the genes in the top 25% of variance for further differential gene expression analysis. Ulcer-immunity-related DEGs between the “ulcer_low-immunity” and “nonulcer_high-immunity” groups were identified as those with fold change > 2 and false discovery rate (FDR) < 0.05. FDR < 0.05 was the significance criterion for cluster-specific genes.

### Gene Set Variation Analysis (GSVA) and Single-Sample Gene Set Enrichment Analysis (ssGSEA)

ssGSEA and GSVA were performed using the R ‘gsva’ package (23). The gene set ‘c2.cp.kegg.v7.4.symbols.gmt’ was downloaded from MSigDB v7.4 for GSVA. For ssGSEA, 24 immune cell signatures (24) were used to describe immune cell populations in individual patients.

### Construction and Validation of the Ulcer Immunity Subtype

To identify prognostic genes with P < 0.05, univariate Cox regression analysis was performed using DEGs identified on comparing the “ulcer_low-immunity” and “nonulcer_high-immunity” groups. Based on 53 prognostic genes, we used the R ‘ConsensuClusterPlus’ package (25) to generate robust clusters of TCGA-SKCM patients. The delta area and cumulative distribution function were used to identify the optimal K value of 3. To validate the subtype in the three merged GEO cohorts (GSE65904, GSE59455, GSE19234), The Nearest Template Prediction (NTP) method provides a convenient way to make classification predictions using only a list of signature genes and a test data set for predictive confidence assessment of gene expression data for each individual patient (26). In our study, the top 200 upregulated of each cluster in TCGA (600 genes in total) were applied to predict the clusters in merged GEO dataset by applying the R ‘MOVICs’ package (27).

### Analysis of Genomic Alterations

Somatic mutations and somatic copy number alternations (CNAs) were downloaded from the GDC using the R ‘TCGAbiolinks’ package (21). The R ‘Maftools’ package (28) was then used to visualize and analyze data pertaining to somatic mutations and CNAs (GISTIC output). GISTIC 2.0 (29) was used to identify significant copy number amplifications and deletions. Chi-square and Fisher’s exact test were used to detect differential mutated genes and differentially copy number gains and losses.

### Quantification of Immune Cell Infiltration

The EPIC (30), CIBERSORT (31, 32), MCPcounter (33), and Quantiseq (34) algorithms were used to estimate the fraction of immune cells based on the transcriptome data (TPM value) of TCGA-SKCM patients.

### Protein-Protein Interaction (PPI) Network Construction and Selection of Hub Genes

The PPIs between differentially expressed genes were searched using the Search Interacting Genes/Proteins (STRING) v.11.5 database with a confidence level of 0.7, and the interaction network was visualized using Cytoscape software. In a PPI network, nodes with higher connectivity are more important for maintaining the stability of the entire network and are therefore considered more relevant to the overall biological process. The radiality method is a topological analysis method that plays an important role in predicting key nodes (35). Using the radiality method of CytoHubba plugin (35) in Cytoscape software, the top 10 genes in the network were screened as hub genes by radiality method.

### Cell Culture, Transient Transfection, RNA Extraction, and Quantitative Real-Time PCR (qRT-PCR)

We herein used the A375 (ATCC, Cellcook Biotechnology, Guangzhou, China) and SK-MEL-28 (ATCC, Cellcook Biotechnology) cell lines. A375 and SK-MEL-28 cells were cultured in DMEM (Gibco, Carlsbad, CA, USA) supplemented with 10% fetal bovine serum (Gibco) and Penicillin Steptomycin (Invitrogen, Carlsbad, CA, USA). For siRNA transfection, A375 and SK-MEL-28 cells were transfected with EIF3B siRNA (RiboBio, Guangzhou, China) using Lipofectamine 3000 (Invitrogen), according to manufacturer instructions. The sequence of siRNAs for targeting EIF3B was as follows: CCTTAGCGTTTGTGGACACTT (EIF3B siRNA1) and CGGGAAGATTGAACTCATCAA (EIF3B siRNA2). Total RNA extraction, purification, and qRT-PCR were performed as previously reported. The sequences of primers used for qRT-PCR were as follows: (1) β-actin: forward primer, CTCGCCTTTGCCGATCC and reverse primer, TTCTCCATGTCGTCCCAGTT and (2) EIF3B: forward primer, CTGGGTGCCTGAAGACAAAGA and reverse primer, CGTTCTTCTGCCAATGGAGC.

### Western Blotting

Western blotting was performed as previously described (36) using primary antibodies against α-tubulin (A11126, Invitrogen) and EIF3B (MA5-36159, Thermo Fisher).

### Transwell Migration and Invasion Assays

For the transwell migration assay, after 16h serum-free starvation, the cells were resuspended in serum-free media; subsequently, 250000 cells in 300 µL serum-free media were seeded into transwell inserts (Corning) with 8-µm pore size. Different treatment media were then added in the lower chamber. For the invasion assay, the inserts were coated with 40 µL matrigel (1 mg/ml, BD Biosciences). The cells were then seeded onto the coated inserts and incubated with different treatment media. After 24 h or 48 h of incubation at 37°C in a CO_2_ incubator, the cells and media were carefully removed from the top of the insert, and the migration or invasion inserts were placed into a clean well. The cells were then fixed in 4% paraformaldehyde and stained with 0.2% crystal violet. After wiping out upper cells in the insert, the cells which grew through the porous membrane were photographed by an inverted light microscope (×100). The relative numbers of migrating and invasive cells were counted by using ImageJ software.

### Analysis of the Response to Immunotherapy

To demonstrate the efficacy of immunotherapy, we integrated two datasets (GSE78220 and GSE91061) of patients with melanoma who had received anti-PD-1 therapy, and only therapy-naïve patients further analyzed. The submap tool (29) was used to predict responsiveness to immunotherapy depending on EIF3B expression.

### ELISA for TGF-β1 Level

Cells were firstly seed at 1×10^5^/well in 12-well plate and the supernatant was collected at 48h. The amount of TGF-β1 in supernatant of EIF3B knockdown cells and scramble control cells were determined by ELISA specific for human TGF-β1(R&D Systems Inc., Minneapolis, MN, USA). The assay was performed following the manufacturer’s instruction. Optical density at 450 nm was measured and the final concentration of TGF-β1 (pg/ml) was calculated according to standard protein curve.

### Development and Validation of the Ulcer-Immunity Related Prognostic Model

Firstly, Multiclass DESeq2 was used to identify 1744 cluster-specific altered genes with an FDR-adjusted P value of < 0.01. Secondly, Univariate cox analysis was performed for the 1744 genes and identify 709 genes with prognostic value in the TCGA SKCM dataset. Thirdly, LASSO (least absolute shrinkage and selection operator) logistic regression with 10-fold cross-validation was used to further reduce candidate genes using the ‘glmnet’ R package (37). Then, multivariate cox analysis (38) was used to further screen genes using the Cox multivariate proportional hazard regression model with a stepwise method (both). The risk score was calculated using the following formula: Risk score = 0.278×EI24 + 0.166×HEYL – 0.09×IFIT3 – 0.18×SNTB1 – 0.115×CSF1R. The risk score for each patient in the TCGA data set and the validation data set was calculated according to this formula. Patients were classified into high-risk and low-risk groups based on the median cut-off for risk scores. Survival analysis was performed using the ‘survival’ R package to assess differences in OS between high- and low-risk groups. To measure the specificity and sensitivity of the prognostic capability of this model, we calculated the area under the curve (AUC) using the R ‘timeRoc’ package (39).

### Statistical Analysis

Kaplan–Meier curve and log-rank test were used to evaluate differences in survival rate between the groups. Univariate and multivariate Cox regression analyses were used to determine prognostic factors. Pearson and Spearman correlation analyses were performed for calculating the correlation coefficient. The unpaired Student’s *t*-test and Mann–Whitney U-test were used for normally and non-normally distributed variables, respectively, so as to compare the two groups. To compare more than two groups, one-way analysis of variance (ANOVA) and Kruskal–Wallis test were applied as parametric and non-parametric methods, respectively. Chi-square and Fisher’s exact tests were used to examine differentially mutated genes and differential copy number gains and losses. The R ‘Maftools’ package (28) aided OncoPrint generation. The R ‘ggplot2’ (40) and ‘ComplexHeatmap’ packages (41) were used for data visualization. All survival curves were generated using the R ‘survival’ (42) and ‘survminer’ (43) packages. Statistical analysis was performed using the R software, and values represent mean ± standard deviation (SD). P < 0.05 indicated statistical significance.

## Results

### Identification of Ulcer-Immunity-Related Prognostic DEGs

**Figure 1:**
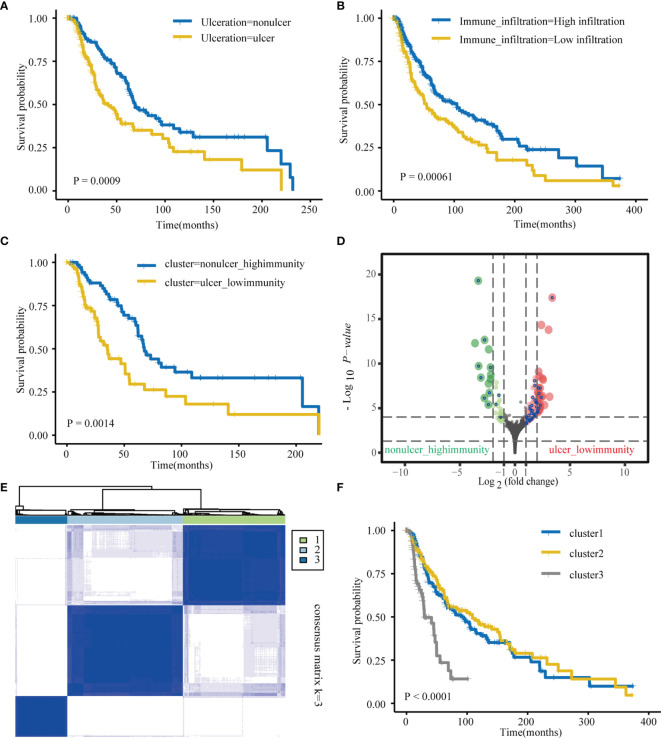
Identification of ulcer-immune–related prognostic DEGs in SKCM. **(A)** Kaplan–Meier analysis showing the association between ulceration and SKCM patient overall survival (OS) in TCGA cohorts. **(B)** Kaplan–Meier analysis showing the association between immune infiltration and SKCM patient overall survival (OS) in TCGA cohorts. **(C)** Kaplan–Meier plot of overall survival (OS) for TCGA SKCM patients in nonulcer-high_immunity and ulcer-low_immunity groups. **(D)** Differential gene (DEGs) between nonulcer-high_immunity and ulcer-low_immunity groups. **(E)** Consensus clustering shows that 3 clusters are the most stable clusters. **(F)** Kaplan–Meier plot of overall survival (OS) for the three subtypes in TCGA SKCM cohort.

First, we explored TCGA-SKCM dataset; **Table S1** details pertaining to the clinical characteristics of TCGA cohort. In case of TCGA database, patients with ulceration had worse prognosis than those with non-ulceration (**Figure 1A**). Tumor samples consist of cancer and many non-tumor cells, such as infiltrating immune and stromal cells, as well as other non-cellular components, which crosstalk with each other and ultimately promote cancer progression (44). For TCGA-SKCM patients, immune cell infiltration was measured *via* ssGSEA using previously published immune cell signatures. The SKCM samples in TCGA were divided into two groups (high and low immunity) depending on the degree of immune cell infiltration (**Supplementary Figure 1**). As with previous reports, the high immune cell infiltration group was associated with better overall survival (OS) than that the low immune cell infiltration group (**Figure 1B**). Based on our clinical-related data, patients were further divided into three groups: “ulcer_low-immunity,” “nonulcer_high-immunity,” and “others.” Survival analyses showed significant differences between the “ulcer_low-immunity” and “nonulcer_high-immunity” groups (P = 0.0014; **Figure 1C**). Further, DESeq2 was used to identify ulcer-immunity-related DEGs on comparing the “ulcer_low-immunity” and “nonulcer_high-immunity” groups. Overall, 158 genes with a Benjamini and Hochberg-corrected P value < 0.05 and fold change > 2 were identified as primary DEGs (**Figure 1D**). We used these genes and OS data available in TCGA-SKCM dataset to perform a survival analysis using the univariate Cox proportional hazards model, and 53 genes were identified as ulcer-immunity-related DEGs. We used the R ‘ConsensusClusterPlus’ package for consistent clustering of genes in TCGA-SKCM dataset, and category identification based on the 53 ulcer-immunity-related DEGs was performed. Using the unsupervised clustering method, three different clusters were finally identified: 177 cases in Cluster 1, 200 in Cluster 2, and 90 in Cluster 3 (**Figure 1E** and **Supplementary Figures 2A, B**). The survival analysis revealed that Cluster 3 had the worst prognosis in comparison to the other clusters (**Figure 1F**). Previous study had shown that TCGA-SKCM sample can be further divided into three molecular subtypes, including ‘immune’, ‘keratin’ and ‘MITF-low’ (45). Furthermore, Alexander Bagaev et al. establish a classifier based on cancer microenvironment and classify tumor samples into four subtypes termed as (1) immune-enriched, fibrotic (IE/F); (2) immune-enriched, non-fibrotic (IE); (3) fibrotic (F); and (4) immune-depleted (D) (46). Interestingly, we found that the proportion of patients with low immune infiltration is highest in cluster 3 (51.2%) and most of the samples in cluster3 were defined as ulceration (78.6%). Moreover, most of the samples in cluster3 were defined as the keratin subtype (79.1%) and immune-depleted subtype (47.7%, **Supplementary Figures 2E–H**). Multiclass DESeq2 was used to identify cluster-specific upregulated genes with an FDR-adjusted P value of < 0.05. Three GEO datasets with usable OS data and clinical information (GSE65904, GSE19234, and GSE59455; **Table S1**) were combined into one metacohort. The three clusters were validated in the merged GEO cohort using the NTP algorithm (FDR < 0.05). Cluster 3 was still associated with the worst prognosis (**Supplementary Figures 2C, D**).

### Functional Annotation and Multiomics Analysis

**Figure 2:**
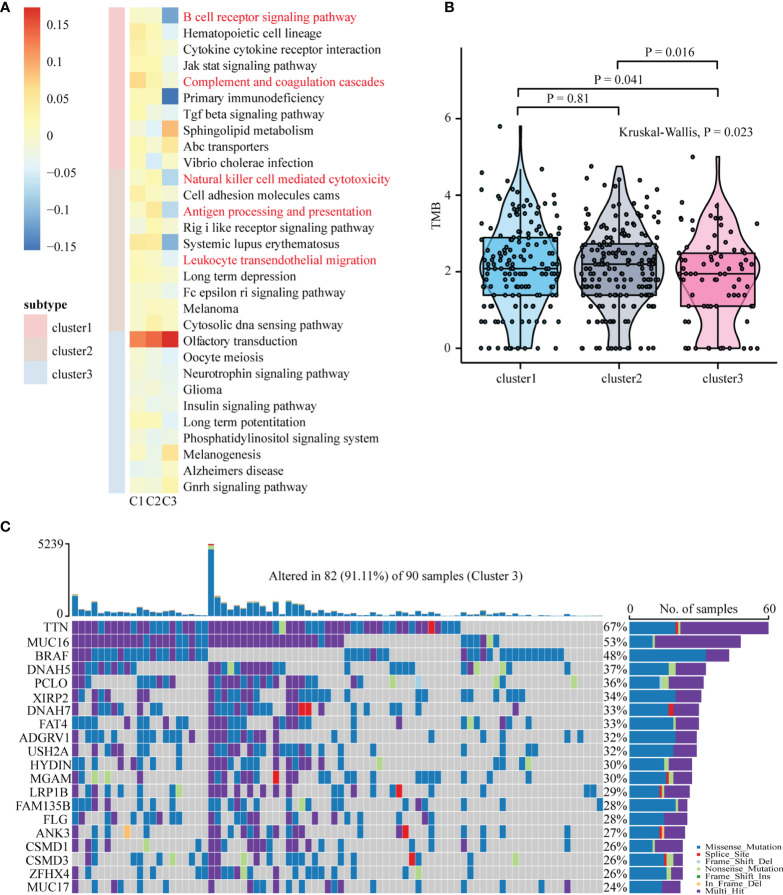
Functional annotation and mutation analysis of SKCM subtypes. **(A)** Top ten enriched pathways for each subgroup were identified by GSVA analysis. **(B)** Differences in tumor mutation burden (TMB/Mb) between different SKCM subtypes. The Kruskal-Wallis test was used to compare the statistical differences between the three subtypes of TCGA-SKCM. TMB/Mb, tumor mutation burden per 1 Mb exome size. **(C)** The waterfall plot showing the top 20 mutated gene of Cluster3. Each column represents a single patient. The upper barplot showed the total tumor mutation burden (TMB), The number on the right shows the mutation frequency of each gene. The bar graph on the right shows the proportion of each mutation type.

**Figure 3:**
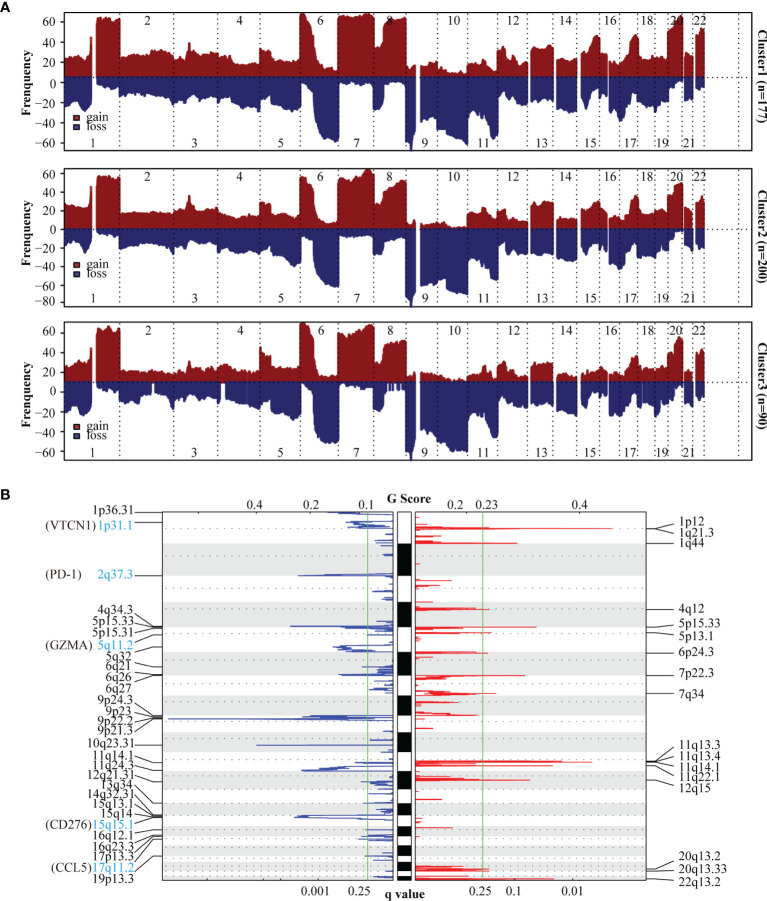
Copy number variation (CNV) analysis of SKCM subtypes. **(A)** The frequency of chromosomal aberrations is shown for the different subtypes. Copy number amplification or deletion is shown in red or blue respectively. **(B)** GISTIC 2.0 analysis showing the amplifications and deletions in Cluster3. Chromosomal locations of peaks of significantly gains (red) and losses (blue) are shown. The q-value, which indicates statistical significance, is displayed at the bottom of graph. Areas with q-values< 0.25 (green lines) are considered significantly altered. The locations of the peak regions of highest copy number change and the known immune checkpoint genes within these peaks are indicated.

To uncover the activation of the signaling pathways in each subtype, we performed GSVA and calculated the enrichment score for KEGG signaling pathways with MSigDB v7.4. We then selected a total of 30 most representative Cluster 1–3 gene sets and created a heatmap showing specific gene sets for each subtype (**Figure 2A**). As evident from the heatmap, in comparison with Clusters 1 and 2, Cluster 3 was characterized by the lack of signals related to the immune system and immune cells. To analyze mutations in each subtype, we created a waterfall chart to depict the top 20 significantly mutated genes (SMGs) in the three clusters (**Figure 2C** and **Supplementary Figures 3A, B**). This chart showed that 50% SMGs (TTN, MUC16, BRAF, PCLO, DNAH5, DNAH7, ADGRV1, LRP1B, ANK3, and CSMD1) were shared by Clusters 1–3. Clusters 1 and 3, but not Cluster 2, shared the top three SMGs: FLG (35% and 28%, respectively), XIRP2 (34% and 34%, respectively), and CSMD3 (33% and 26%, respectively). Clusters 2 and 3, but not Cluster 1, also shared the top three SMGs: MGAM (38% and 30%, respectively), HYDIN (34% and 30%, respectively), and USH2A (34% and 32%, respectively). Moreover, Cluster 3 showed four unique top SMGs: FAT4 (33%), FAM135B (28%), ZFHX4 (26%), and MUC17 (24%). In a recent prospective study, higher tumor mutation burden (TMB) was found to be associated with a better response to immunotherapy (47). The tumor mutations in Cluster 2 samples were slightly higher than those in Cluster 1 samples, and the severity of TMB in Cluster 1 and 2 samples was higher than that in Cluster 3 samples (**Figure 2B**). We then used GISTIC to analyze data related to somatic CNAs to identify areas that were repeatedly amplified and deleted among Clusters 1–3, and found an obvious similarity among chromosomal aberrations in the three clusters (**Figure 3A** and **Supplementary Figure 4A**). The similarity between Clusters 2 and 3 was stronger than that between Clusters 1 and 3. In total, 29 focal deletion and 17 focal amplification peaks were detected in Cluster 3, while 41 and 44 focal loss and 43 and 49 focal gain peaks were detected in Clusters 1 and 2, respectively (**Figure 3B** and **Supplementary Figures 4B, C**). Next, we examined the frequency of amplification or deletion for immune checkpoint genes in each subtype. Many immune checkpoint genes were deleted in Cluster 3 (PD-1, GZMA, CD276, CCL5, and VTCN1), while several were amplified in Cluster 1 (CD4, CD274, CD276, and HLA gene family) and Cluster 2 (CD274 and PDCD1LG2; **Figure 3B** and **Supplementary Figures 4B, C**).

### Immune Landscape of Patients With Melanoma in Different Clusters

**Figure 4:**
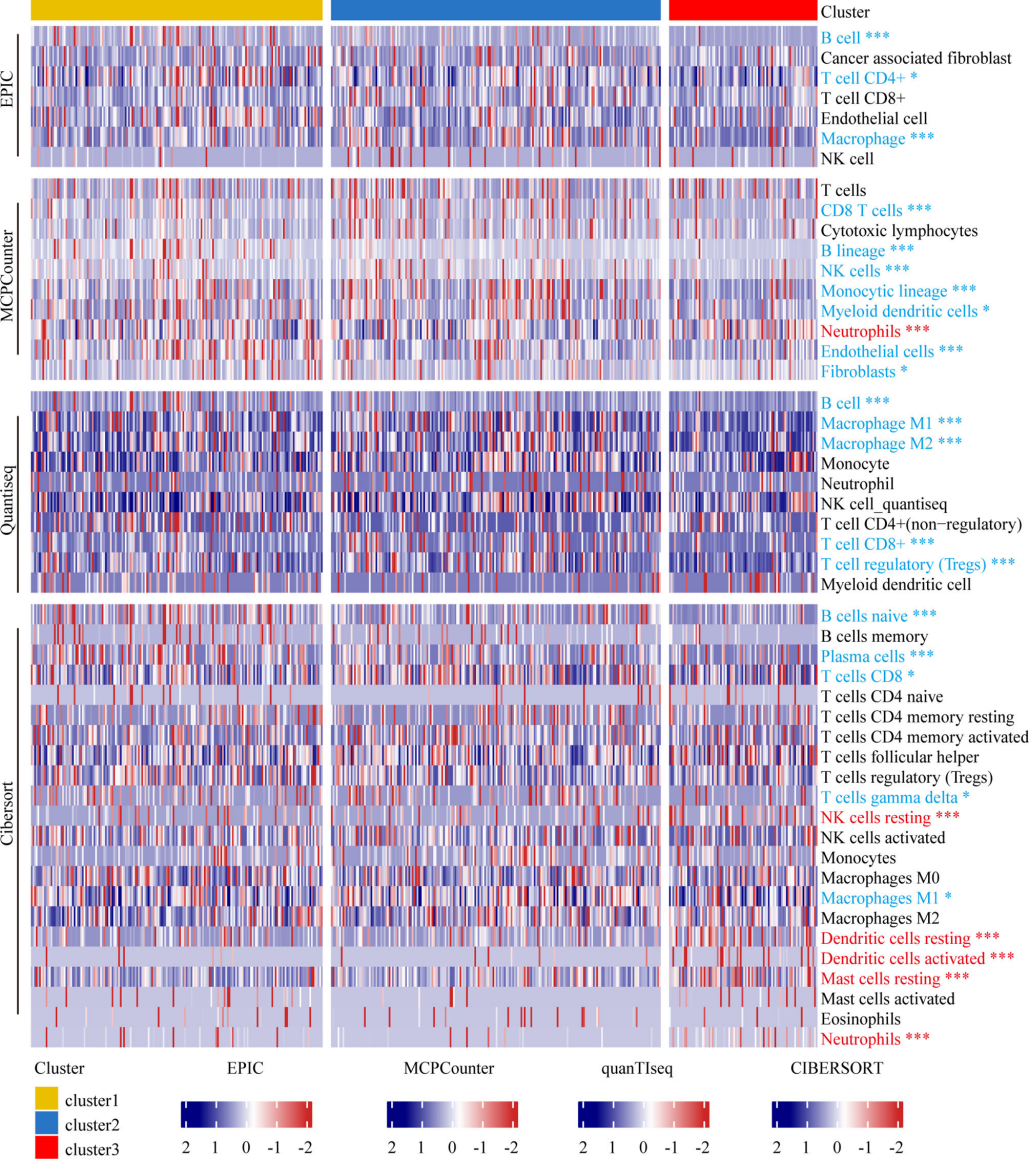
Immune landscape of melanoma patients within different clusters. Heatmap representing the differences in immune cell infiltration between different subtypes based on EPIC, MCPcounter, Quantiseq, and Cibersort algorithms. The Kruskal-Wallis test was used to compare the statistical differences. “*” indicates p-value ≤ 0.05, “***” indicates p-value ≤ 0.001.

Our data indicated that Cluster 3 was negatively associated with immune-related pathways and mainly immune-depleted subtype. We investigated the heterogeneity of immune cell infiltration in the three clusters based on the EPIC, MCPcounter, Quantiseq, and CIBERSORT algorithms. Tumor-infiltrating immune cells in 467 TCGA melanoma patients was showed in the heatmap (**Figure 4**). B cells infiltration in Cluster 3 showed a significantly decrease according to all the four algorithms; further, according to three algorithms, macrophages and CD8+ T cells infiltration in Cluster 3 was significantly lower. Collectively, these results revealed that Cluster 3 had a different immune phenotype than Clusters 1 and 2, with Cluster 3 showing less immune cell infiltration and less immune activation.

### Differences in Immune-Related Genes and Response to Anti-PD-1/L1 Immunotherapy

**Figure 5:**
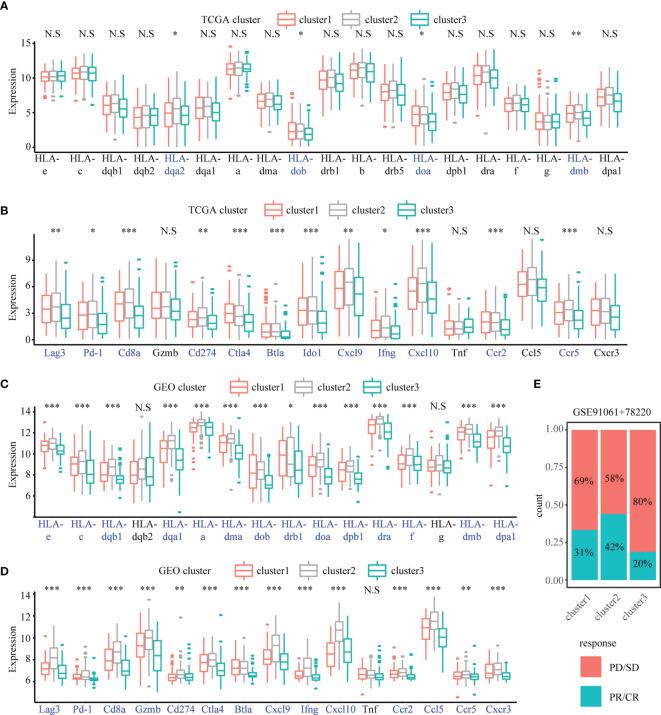
The difference of immune-related gene and response of anti-PD-1/L1 immunotherapy in the SKCM subtypes. Boxplots showing the differences in the expression of immune-related genes and immune checkpoint genes between different subtypes in TCGA cohort **(A, B)** and GEO cohorts **(C, D)** The Anova test was used to compare the statistical differences. “*” indicates p-value ≤ 0.05, “**” indicates p-value ≤ 0.01, “***” indicates p-value ≤ 0.001, N.S indicates not significant (p > 0.05). **(E)** The percentage of patients with response to anti-PD-1 immunotherapy in different subtypes. SD, stable disease; PD, progressive disease; CR, complete response; PR, partial response.

We investigated the expression of HLA family genes and immune checkpoint markers among the three subtypes in TCGA and GEO datasets. In case of HLA family genes, the expression levels of HLA-DQA2, HLA-DOB, HLA-DOA, and HLA-DMB in Cluster 3 of TCGA dataset were less than the other two clusters; there was no significant difference (ANOVA test, P < 0.05) in the expression level of other HLA family genes among the three clusters (**Figure 5A**). However, in the GEO dataset (GSE65904 + GSE19234 + GSE59455), all HLA family genes, except HLA-G, HLA-DQB2, exhibited downregulated expression levels (ANOVA, P < 0.05) in Cluster 3 relative to the other two clusters (**Figure 5C**). We then assessed immune checkpoint markers associated with antigen presentation, cell surface receptors, co-inhibition, ligands, and cell adhesion. In comparison with Clusters 1 and 2, Cluster 3 in TCGA dataset showed reduced expression of all immune checkpoint markers, except GZMB, TNF, CCL5, and CXCR3. Besides, in the GEO dataset, Cluster 3 showed reduced expression of all immune checkpoint markers (**Figures 5B, D**). Furthermore, we combined GSE78220 and GSE91061 with available response to immunotherapy and used the specific gene of three clusters for analyses with the NTP algorithm to predict the subtypes in the two GEO datasets (FDR < 0.05; **Supplementary Figure 5C**). We also grouped the treatment response into a binary model and found that the percentage of patients with stable/progressive disease group in Cluster 3 was higher than the other two clusters (**Figure 5E**).

### EIF3B as a Cluster 3-Specific Hub Gene and Its Role in Melanoma Progression

**Figure 6:**
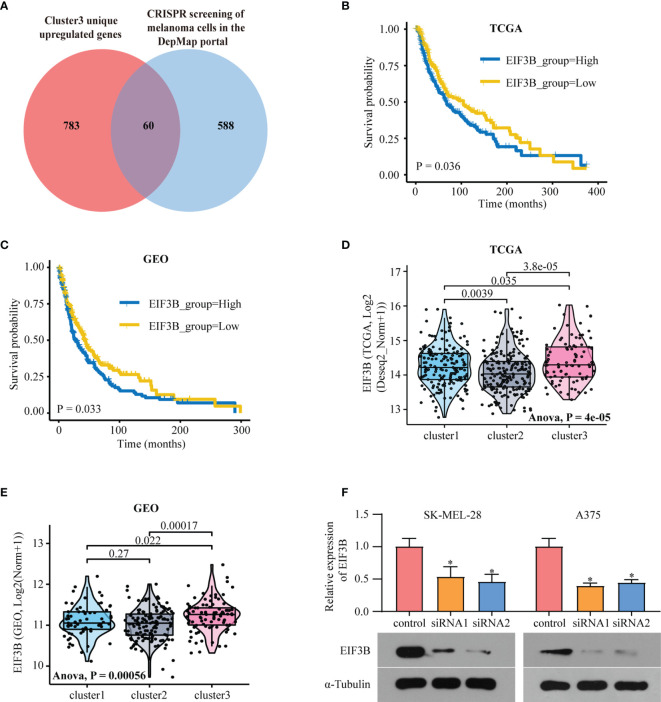
EIF3B is a poor prognostic marker in melanoma. **(A)** Venn diagram shows the intersection of Cluster3 specific upregulated genes and the genes which are essential to melanoma human cell line growth using the DepMap database. **(B)** Kaplan–Meier analysis showing the association between the expression of EIF3B and SKCM patient overall survival (OS) in TCGA cohort **(B)** and GEO cohorts **(C)**, the Log-rank test was used to access the statistical differences. The differences of EIF3B expression between different subtypes in TCGA dataset **(D)** and GEO datasets **(E)**, the Anova test was used to calculate the statistical differences. **(F)** RT-qPCR and Western blot were used to verify the efficiency of EIF3B knockdown in SK-MEL-28 and A375 cell line. ‘*’ indicates p-value ≤ 0.05.

**Figure 7:**
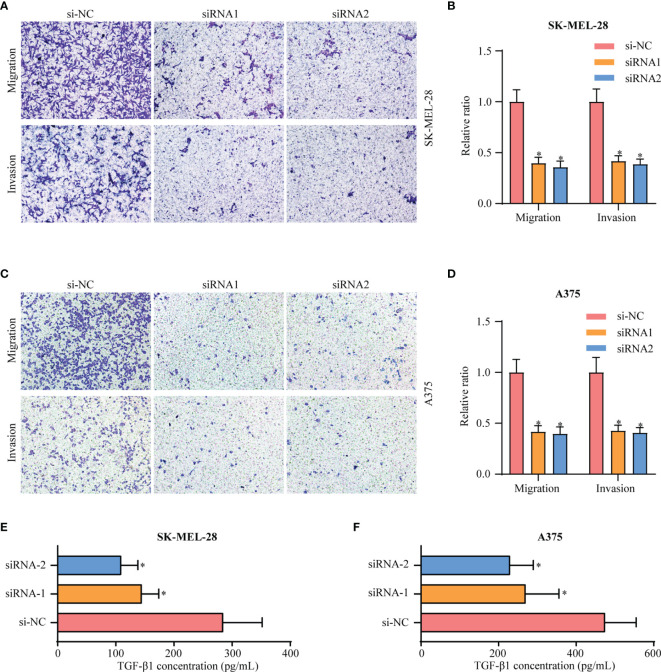
Knockdown of EIF3B inhibits the migration and invasion of melanoma cell lines *in vitro*. **(A, B)** silencing EIF3B suppressed migration and invasion abilities in SK-MEL-28 cells. **(C, D)** silencing EIF3B suppressed migration and invasion abilities in A375 cells. **(E, F)** silencing EIF3B suppressed reduces the ability of SK-MEL-28 and A375 cells to secrete TGF-β1. Error bars represent the mean ± S.D. of three independent experiments. “*” indicates p-value ≤ 0.05.

In comparison with Clusters 1 and 2, Cluster 3 showed the worst prognosis and responsiveness to immunotherapy. According to our results, Cluster 3 can be used as an independent prognostic factor in both TCGA and GEO databases (**Table S2**). We then aimed to identify a hub gene in Cluster 3 with a key role in melanoma progression. To discover the main genes responsible for SKCM growth, CRISPR-based genome-wide loss-of-function screening was employed (DepMap, https://depmap.org/portal/download/), and 648 genes in the SKCM cell line were found to be important for survival. Among these 648 candidate genes, 60 were specific to Cluster 3 (**Figure 6A**). **Supplementary Figures 5A, B** shows the expression levels of these 60 genes in all three clusters. We then explored the PPI network based on these 60 Cluster 3-specific genes and the top 10 genes in the network were screened as hub genes by radiality method (35). (**Supplementary Figure 5D**). Survival analysis revealed that among these top 10 genes, only EIF3B had prognostic significance both in TCGA and GEO databases; higher expression level of EIF3B was associated with worse prognosis in patients with melanoma (**Figures 6B, C**). We also found that Cluster 3 showed the highest expression of EIF3B (**Figures 6D, E**). EIF3B has been previously reported to play a vital role in the progression of several types of cancers (17, 18, 48), but its effectiveness in melanoma remains to be reported. To further study the effects of EIF3B on the biofunction of melanoma cells *in vitro*, we constructed stable knockdown A375 and SK-MEL-28 cell lines that expressed EIF3B-specific siRNAs (**Figure 6F**). As showed in **Figures 7A–D**, the results of transwell migration assay and invasion assay showed that the relative number ratios of migrating and invasive cells were significantly decreased in EIF3B knockdown cells compared with si-NC cells. It indicated that EIF3B knockdown could suppress migration and invasion abilities in A375 and SK-MEL-28 cells.

### High Expression of EIF3B Was Correlated With Low Immune Cell Infiltration and Could Predict the Clinical Benefits of Immune Checkpoint Blockade

**Figure 8:**
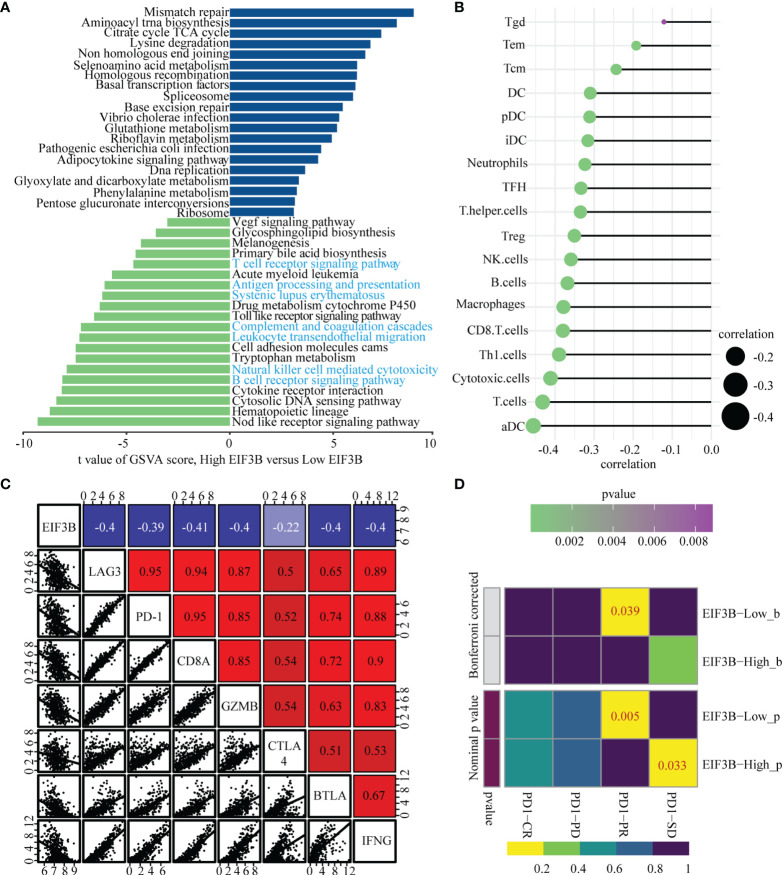
High expression of EIF3B correlates with low immune cells infiltration and predict the clinical benefit of ICB. **(A)** Top twenty enriched pathways in high and low EIF3B expression group were identified by GSVA analysis. **(B)** The graph showing the correlation of EIF3B expression and immune cell signature based on ssGSEA analysis output. The Spearman rank correlation test was used to calculate the correlation coefficients. **(C)** Correlations among EIF3B, LAG3, PD-1, CD8A, GZMB CTLA4, BTLA and IFNG levels in TCGA SKCM dataset. The Pearson correlation test was used to calculate the correlation coefficients. **(D)** The submap analysis shows that the lower EIF3B group in the GEO cohort is more sensitive to anti-pd-1 treatment. P-values were obtained after being adjusted by the Boferroni method.

To explore EIF3B-associated biological functions in melanoma, we performed GSVA using TCGA-SKCM dataset. High expression of EIF3B was associated with significantly downregulated immune-related pathways, such as the T cell receptor pathway, B cell receptor pathway, antigen processing and presentation, and leukocyte transendothelial migration (**Figure 8A**). Based on these finding, we believe that EIF3B plays a key role in the immune response in melanoma. We used the Spearman order correlation method to detect the correlation between immune system cells and EIF3B in TCGA dataset. EIF3B expression was found to be negatively correlated with immune cell levels, including those of effector cells, in immunotherapy (**Figure 8B**). Furthermore, we investigated the correlation between seven immune checkpoint markers (LAG3, PD-1, CD8A, GZMB, CTLA-4, BTLA, and IFNG) and EIF3B expression in TCGA and GEO datasets, and found that EIF3B expression was negatively correlated with all of them in both datasets (**Figure 8C** and **Supplementary Figure 6A**). These results suggested that high expression of EIF3B leads to deficient proinflammatory immune cell infiltration and might predict a worse response to immunotherapy. We then performed submap analyses to evaluate the response of melanoma patients with high and low EIF3B expression to anti-PD-1 immunotherapy. Interestingly, patients with low EIF3B expression showed partial response to anti-PD-1 immunotherapy (**Figure 8D**). The effectiveness of cancer immunotherapy is highly dependent on the development and activation of the steps in the cancer immunity cycle (49). Therefore, we further analyzed the correlation between EIF3B expression and anti-cancer immunity cycle and found EIF3B expression was negatively correlated with all the steps in the cycle (**Supplementary Figure 6B**). Furthermore, the ELISA results showed that the concentration of TGF-β1 in supernatant was significantly decreased in EIF3B knockdown cells compared with si-NC cells (**Figures 7E, F**). These results suggested that EIF3B plays a suppressive role in melanoma immune response and immunotherapy.

### Construction of the Ulcer-Immunity Related Prognostic Model

**Figure 9:**
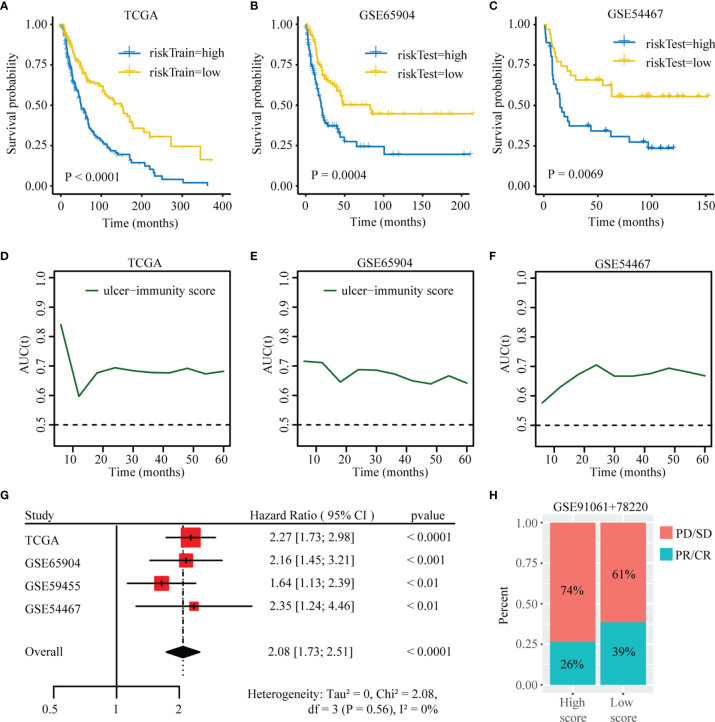
Construction of the ulcer-immunity related prognostic model. **(A)** Kaplan–Meier analysis showing the association between risk score and SKCM patient overall survival (OS) in TCGA cohorts. Kaplan–Meier analysis showing the association between risk score and SKCM patient overall survival (OS) in GSE65904 **(B)** and GSE54467 **(C)**. Time-dependent AUC value in TCGA **(D)**, GSE65904 **(E)** and GSE54467 **(F)**. **(G)** Meta-analysis of the prognostic values of ulcer-immunity related risk score in melanoma. **(H)** The percentage of patients with response to anti-PD-1 immunotherapy in high-risk and low-risk groups. SD, stable disease; PD, progressive disease; CR, complete response; PR, partial response.

We firstly performed Lasso logistic regression and stepwise multivariate cox analysis to constructed a prognostic model based on cluster specific altered genes with prognostic value in the TCGA SKCM dataset (**Supplementary Figures 7A, B**). The risk score was calculated as follows: Risk score = 0.278×EI24 + 0.166×HEYL - 0.09×IFIT3 - 0.18× SNTB1 - 0.115×CSF1R. Then, patients in TCGA dataset were divided in high- and low-risk groups based on their risk score. We found that the expression of EIF3B was higher in high-risk groups (**Supplementary Figure 8A**) and the patients in cluster3 had a higher risk score (**Supplementary Figures 8B, C**). Kaplan-Meier survival analysis was performed on the training and testing datasets to assess the predictive power of our ulcer-immunity related signatures. In the TCGA dataset, high-risk patients exhibited worse OS compared to low-risk patients (P < 0.001, **Figure 9A**). A similar result was observed in the testing datasets (GSE65904, GSE54467, GSE59455) (**Figures 9B, C** and **Supplementary Figure 8D**). Meantime, time-dependent AUC and AUC at 1, 2, 3, and 5 years suggested that the ulcer-immunity related score had a significant value in predicting the OS of melanoma patients in the TCGA and GEO datasets (**Figures 9D–F** and **Supplementary Figures 8E–I**). To examine the combined prognostic value of all training and testing datasets, we performed a prognostic meta-analysis. Results showed that the ulcer-immunity related score was a significant risk factor for overall survival of patients with melanoma (combined HR = 2.08, 95% CI = 1.73-2.51, P < 0.0001, **Figure 9G**). Furthermore, we calculated the risk score of the patients in GSE78220 and GSE91061 datasets with available response to immunotherapy, and it was found that the percentage of stable/progressive disease in high-risk patients was higher than that in low-risk patients (**Figure 9H**).

## Discussion

Recent research has enhanced our understanding of the biological and molecular characteristics of melanoma. However, the demand for clinically relevant classification of melanoma to guide treatment options remains to be met. Considering that there exists strong evidence regarding the importance of ulceration and immune checkpoint inhibitors in the prognosis of melanoma, we identified a prognostic model based on ulceration and immune related genes, providing clues for prognosis prediction of melanoma.

Ulceration has been recognized as a significant prognostic factor related to increased risk for recurrence and mortality in melanoma. Balch et al. and Andrea Maurichi et al. reported a significant effect of ulceration concerning overall survival or disease-free survival for melanoma patients (9, 50). Moreover, immune cell infiltration is an independent predictor of overall survival in melanoma (51). In the previous clinical study, it was proved that melanoma patients with a pronounced tumor-infiltrating lymphocyte grade had an excellent prognosis (4). In our study, we also found that patients with ulceration and low immunity had worse overall survival than those without ulceration in TCGA cohort. Furthermore, it was proved that CD8+ T cells was lower in patients with an ulcerated melanoma and tended to correlate with longer overall survival (14). However, an ulcer-immunity related prognostic model in melanoma has not yet been studied.

Based on the effects of ulceration and immune cell infiltration in melanoma, we inferred that a combination of ulceration and immune related genes could be applied to establish a prognostic model which can provide predictive value in melanoma. In the TCGA cohort, we identified 53 ulcer-immunity prognostic genes between the “ulcer_low-immunity” and “nonulcer_high-immunity” groups, and revealed three ulcer-immunity related subtypes in melanoma. Of them, Cluster 3 showed the worst prognosis both in TCGA and GEO datasets, which suggesting that the identified ulcer-immunity prognostic DEGs can be used to establish clinically relevant classification of melanoma. In addition, we also found that Cluster 3 lacked immune-related signaling pathways, including B cell receptor signaling pathway, complement and coagulation cascades, leukocyte transendothelial migration, natural killer cell mediated cytotoxicity and so on. These immune-related signaling pathways are important features in predicting prognosis and related to cancer progression, immunotherapy response and recurrence (52–55).

Tumor mutation burden (TMB) is another useful biomarker for measuring the number of mutations in a cancer and for identification of patients that will benefit from immunotherapy (47). The more mutations, the more neo-antigens and the higher the chances that one or more of those autologous neoantigens will develop immunogenicity and trigger a T cell response (56). In our study, it was showed that Cluster 3 had the lowest TMB compared to the other subtypes in the TCGA cohort, indicating that patients in Cluster 3 could have the weaker responsiveness to immunotherapy compared to Cluster 1 and 2. In addition, the expression levels of immune checkpoint and immune-related genes are reportedly related to the response of melanoma to checkpoint blockade immunotherapy. We further explored that in each subtype, only Cluster 3 had loss of immune checkpoint genes including VTCN1, PD-1, GZMA, CD276 and CCL5. This suggested that patients in Cluster 3 may have a stronger immunosuppressive effect and may be more refractory to immunotherapy.

The tumor microenvironment is a complex network of interactions between tumor cells, immune cells and stromal cells (57). It has proved that immune infiltration is statistically correlated with more favorable prognosis (4). In melanoma, the high intensity of melanoma-infiltrating CD8+ T cells (58) and B cells (59, 60) are associated with positive clinical outcome of immunotherapy-treated patients. In our study, we measured immune cell composition in each subtype and found that Cluster 3 showed less immune cell infiltration, including T and B cells, which could be considered as non-inflamed tumor subtype. Given the strong evidential basis that loss of human leukocyte antigen (HLA) gene, encoding cell surface antigen-presenting proteins, plays an essential role in tumor immune escape and may contribute to immunotherapy resistance (61). And the activation of immune checkpoints mechanism in cancers plays an important role in suppressing the anti-tumor immune response (7, 62, 63). Therefore, we identified the expression of immune checkpoint markers and HLA family genes in each subtype. It was found that only Cluster 3 showed reduced expression of immune checkpoint markers and HLA family genes in TCGA and GEO cohorts. Besides, we used the NTP algorithm to predict the response of each subtype of immunotherapy and found that Cluster 3 included a greater proportion of non-responders to PD-1 immunotherapy, although there was no statistical difference. The lack of statistical difference could be due to the small sample size. Therefore, combined with the characteristics in each subtype, it could be confirmed that Cluster 3 can serve as an independent prognostic factor in both TCGA and GEO datasets. Moreover, we constructed a prognostic model based on these three clusters specific altered gene. We established and verified a prognostic risk signature using five ulcer-immunity related genes (CSF1R, EI24, HEYL, IFIT3, SNTB1). This study had the advantage of assessing the performance of the ulcer-immunity related risk score model as it has been validated in four independent datasets. Time-dependent AUC showed that ulcer-immunity related risk score had a good accuracy in predicting the OS in TCGA and four GEO datasets. Taken together, these results indicated that ulcer-immunity related prognostic model could have clinical applications in melanoma.

To explore a potential therapeutic drug target in Cluster 3, we identified Cluster 3 specific genes with an influence on melanoma progression. These specific genes were then intersected with candidate genes pivotal for melanoma cell growth in the CRISPR screening data from the DepMap database. Among these intersected genes, the top 10 hub genes were selected for OS analysis in TCGA and GEO databases. We thus found that only EIF3B expression was significantly correlated with OS in both databases. High EIF3B expression is associated with the development of various cancers, such as gastric cancer, prostate cancer, and osteosarcoma. Wang et al. (17), and Ma et al. (18) proved that upregulation of EIF3B promoted tumor occurrence and metastasis/colonization. Choi et al. found that EIF3B was essential for osteosarcoma growth *via* regulating TNFRSF21 expression (64). However, its role in melanoma remains unclear. This prompted us to further investigate the role of EIF3B in melanoma progression.

Our *in vitro* experiments revealed that EIF3B knockdown significantly inhibited the migration and invasion of melanoma cells. We further found that EIF3B expression was associated with decreased immune-related pathways and lesser immune cell infiltration. Moreover, EIF3B expression was negatively correlated with some immune checkpoint genes, such as PD-1, GZMB, and CTLA4, which may provide essential information for the development of certain drugs. Recent studies have found that TGF-β1 is essential for immunosuppression in the tumor microenvironment and it also plays an important role in poor response to cancer immunotherapy (65, 66). In our study, we found that the level of TGF-β1 in supernatant was significantly decreased after EIF3B knockdown. These findings suggest that EIF3B could play an important role in melanoma immunotherapy response. In addition, submap analyses supported that low EIF3B expression was correlated with partial response to anti-PD1 therapy, and correlation analyses showed that EIF3B expression was negatively correlated with the steps in the cancer immunity cycle. To summarize, as the hub gene in Cluster 3, EIF3B was found to promote melanoma invasion and progression, which also potentially explains the immunosuppressive characteristic of Cluster 3. As with all studies, even this study has some limitations. First, we did not distinguish between primary and metastatic melanoma when establishing our prognostic model. Second, the signaling pathways and molecular mechanisms underlying the role of EIF3B in the regulation of melanoma progression demand further elucidation. Thirdly, in this study, we only focus on studying EIF3B at RNA level, so it would be interesting to study its prognostic role at protein level in the future. Finally, the relationship between EIF3B and its immunosuppressive role in melanoma needs to be further studied in *in vivo* models.

In conclusion, we developed and verified ulcer-immunity related prognostic model which provides predictive value in melanoma. Further, we verified the potential role of EIF3B in the OS of patients with melanoma and response to immunotherapy. We believe that our ulcer-immunity related prognostic model can widen our understanding of the biology of melanoma and prognosis prediction and that EIF3B can act as a promising therapeutic drug target in melanoma treatment.

## Data Availability Statement

The original contributions presented in the study are included in the article/**Supplementary Material**. Further inquiries can be directed to the corresponding author.

## Author Contributions

ZW, KL, and ES designed the study. ZW and ES wrote the manuscript. KL and JH performed the *in vitro*. SX supervised the study and edited the manuscript. All authors contributed to the article and approved the submitted version.

## Funding

This work was supported by the China Scholarship Council (CSC202006380042, CSC202108080048).

## Conflict of Interest

SX is employed by Shenzhen Mindray Bio-Medical Electronics Co., Ltd.

The remaining authors declare that the research was conducted in the absence of any commercial or financial relationships that could be construed as a potential conflict of interest.

## Publisher’s Note

All claims expressed in this article are solely those of the authors and do not necessarily represent those of their affiliated organizations, or those of the publisher, the editors and the reviewers. Any product that may be evaluated in this article, or claim that may be made by its manufacturer, is not guaranteed or endorsed by the publisher.

## Supplementary Material

Supplementary Figure 1 Immune landscape of melanoma. Unsupervised clustering of 467 patients from the TCGA cohort (n = 467) using ssGSEA and ESTIMATE analysis based on 24 reported immune cell signatures. ImmuneScore, StromalScore, ESTIMATEScore, TumorPurity, clark level, gender, metastasis, as well as survival status were annotated in the lower panel. Euclidean distance and Ward linkage were used to implement unsupervised hierarchical clustering. Two distinct immune infiltration clusters, here termed as high infiltration and low infiltration, were identified.

Supplementary Figure 2 Define the stable cluster of concensus clustering and validate it from GEO datasets. **(A, B)** The cumulative density functions (CDF) was established for a range from 2 to 6 consensus clusters. The delta curve represents CDF progression graph and shows the relative change in the area under the CDF curve. **(C)** Predicted classification of merged GEO cohorts RNA-Seq data was performed using the TCGA-derived cluster specific upregulated genes and Nearest Template Prediction (NTP) algorithm. **(D)** Kaplan–Meier plot of overall survival (OS) for the three subtypes in merged GEO cohort. **(E)** The percentage of patients with different cancer microenvironment subtypes in different cluster. **(F)** The percentage of patients with different TCGA melanoma subtypes in different cluster. **(G)** The percentage of patients with different immune infiltration in different cluster. **(H)** The percentage of patients with ulceration in different cluster. D, immune-depleted; F, fibrotic; IE, immune-enriched, non-fibrotic; IE/F, immune-enriched, fibrotic.

Supplementary Figure 3 mutation analysis of SKCM subtypes. **(A)** The waterfall plot showing the top 20 mutated gene of Cluster1. **(B)** The waterfall plot showing the top 20 mutated gene of Cluster2. Each column represents a single patient. The upper barplot showed the total tumor mutation burden (TMB), The number on the right shows the mutation frequency of each gene. The bar graph on the right shows the proportion of each mutation type.

Supplementary Figure 4 Copy number variation (CNV) analysis of SKCM subtypes. **(A)** The differences in gistic score among the three subtypes. Copy number amplification or deletion is shown in red or blue respectively. **(B)** GISTIC 2.0 analysis showing the amplifications and deletions in Cluster1. **(C)** GISTIC 2.0 analysis showing the amplifications and deletions in Cluster2. Chromosomal locations of peaks of significantly gains (red) and losses (blue) are shown. The q-value, which indicates statistical significance, is displayed at the bottom of graph. Areas with q-values< 0.25 (green lines) are considered significantly altered. The locations of the peak regions of highest copy number change and the known immune checkpoint genes within these peaks are indicated.

Supplementary Figure 5 EIF3B as a hub gene of Cluster3 specific gene. **(A, B)** Boxplots showing that expression of 60 intersected genes (Cluster3 specific upregulated genes and essential genes in the DepMap database) across the three subtypes. **(C)** Predicted classification of merged GEO (GSE91060+78220) anti-PD-1 immunotherapy dataset was performed using the TCGA-derived cluster specific upregulated genes and Nearest Template Prediction (NTP) algorithm. **(D)** The networks of hub genes in the 60 intersected genes (Cluster3 specific upregulated genes and essential genes in the DepMap database) were identified using the Radiality method.

Supplementary Figure 6 Roles of EIF3B in predicting immune response. **(A)** Correlations among EIF3B, LAG3, PD-1, CD8A, GZMB CTLA4, BTLA and IFNG levels in merged GEO dataset. **(B)** Correlations between EIF3B and the steps of the cancer immunity cycle in TCGA SKCM cohort. The Pearson correlation test was used to calculate the correlation coefficients.

Supplementary Figure 7
**(A)** 10-fold cross-validation to select tuning parameters for LASSO models. **(B)** Forest plot of hazard ratios from multivariable Cox proportional hazard regression model.

Supplementary Figure 8
**(A)** The differences of EIF3B expression between high-risk and low-risk groups in TCGA dataset, the Student-t test was used to calculate the statistical differences. **(B)** The differences of risk score between different subtypes in TCGA dataset, the Kruskal-Wallis test was used to calculate the statistical differences. **(C)** The percentage of patients with different risk score in different cluster. **(D)** Kaplan–Meier analysis showing the association between risk score and SKCM patient overall survival (OS) in GSE59455. **(E)** Time-dependent AUC value in GSE59455. **(F–I)** AUC values for 1-, 2-, 3- and 5-year OS in TCGA and GSE65904, GSE59455, GSE54467..
