# Supplementary figures and images for: Establishing a Prognostic Model Based on Ulceration and Immune Related Genes in Melanoma Patients and Identification of EIF3B as a Therapeutic Target
Source: Front Immunol. 2022 Feb 22;13:824946. doi: 10.3389/fimmu.2022.824946 (PMC8901887; doi:10.3389/fimmu.2022.824946)

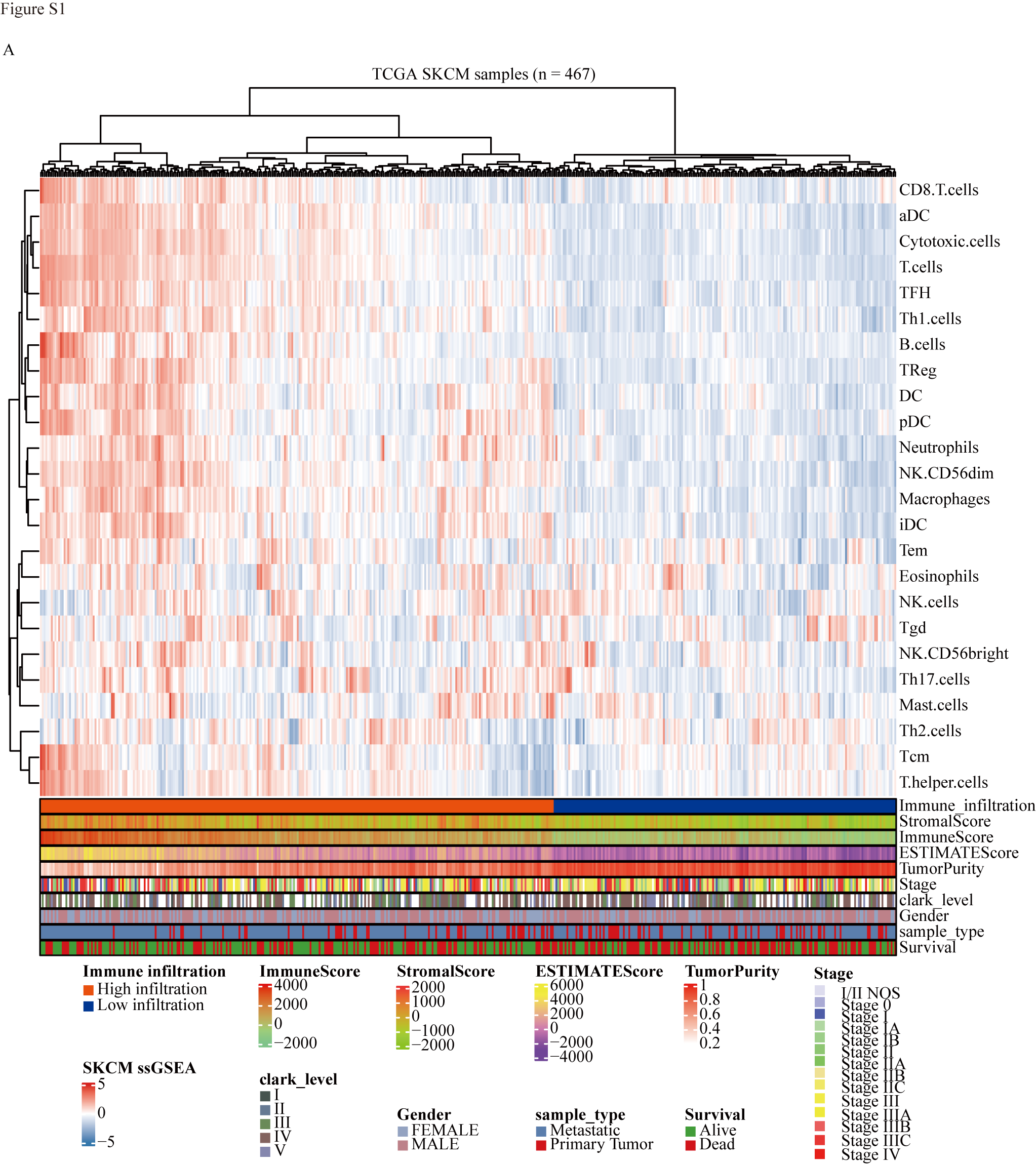

Supplement: Supplementary Figure 1 — Immune landscape of melanoma. Unsupervised clustering of 467 patients from the TCGA cohort (n = 467) using ssGSEA and ESTIMATE analysis based on 24 reported immune cell signatures. ImmuneScore, StromalScore, ESTIMATEScore, TumorPurity, clark level, gender, metastasis, as well as survival status were annotated in the lower panel. Euclidean distance and Ward linkage were used to implement unsupervised hierarchical clustering. Two distinct immune infiltration clusters, here termed as high infiltration and low infiltration, were identified. [file Image_1.tif]

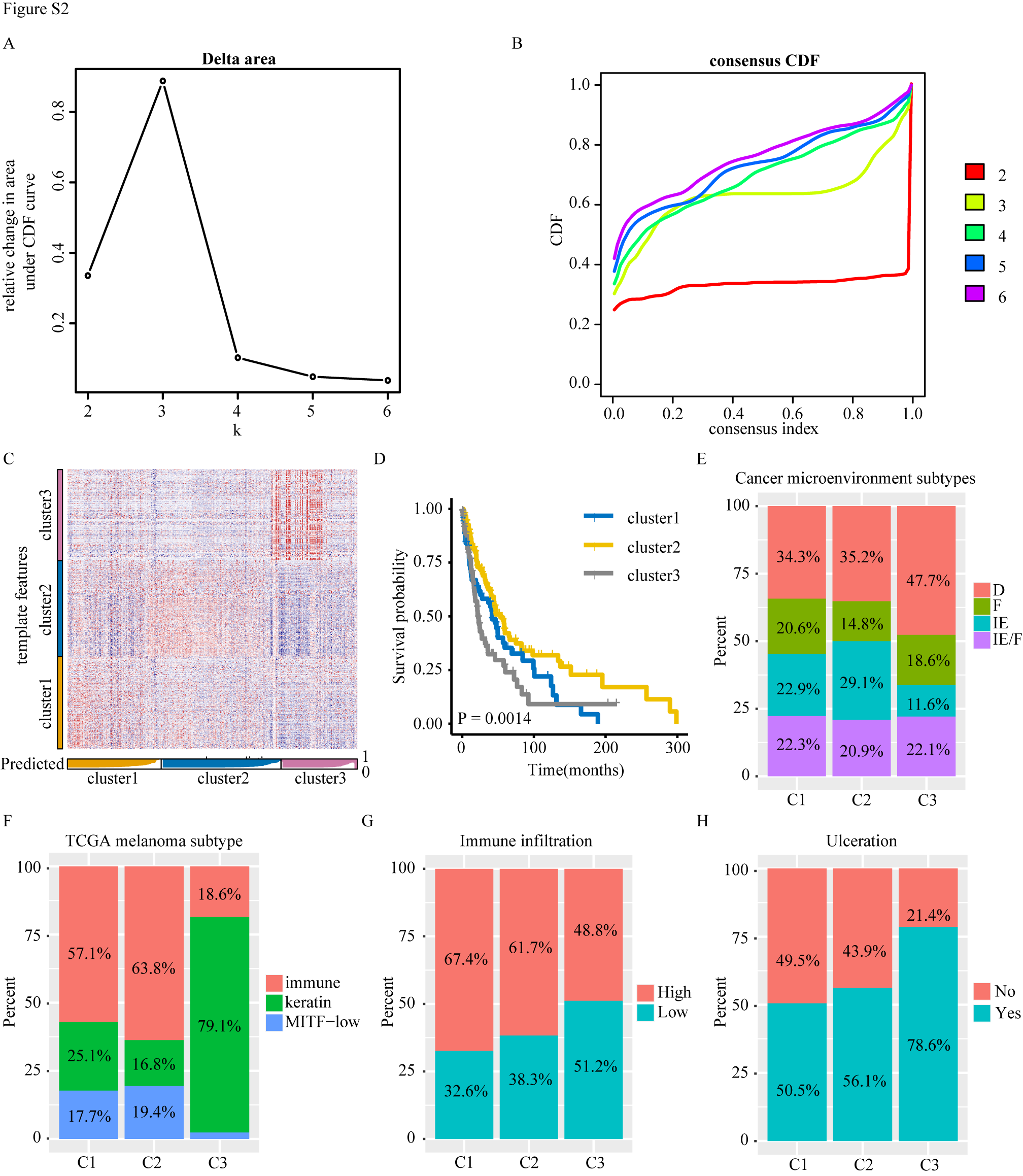

Supplement: Supplementary Figure 2 — Define the stable cluster of concensus clustering and validate it from GEO datasets. (A, B) The cumulative density functions (CDF) was established for a range from 2 to 6 consensus clusters. The delta curve represents CDF progression graph and shows the relative change in the area under the CDF curve. (C) Predicted classification of merged GEO cohorts RNA-Seq data was performed using the TCGA-derived cluster specific upregulated genes and Nearest Template Prediction (NTP) algorithm. (D) Kaplan–Meier plot of overall survival (OS) for the three subtypes in merged GEO cohort. (E) The percentage of patients with different cancer microenvironment subtypes in different cluster. (F) The percentage of patients with different TCGA melanoma subtypes in different cluster. (G) The percentage of patients with different immune infiltration in different cluster. (H) The percentage of patients with ulceration in different cluster. D, immune-depleted; F, fibrotic; IE, immune-enriched, non-fibrotic; IE/F, immune-enriched, fibrotic. [file Image_2.tif]

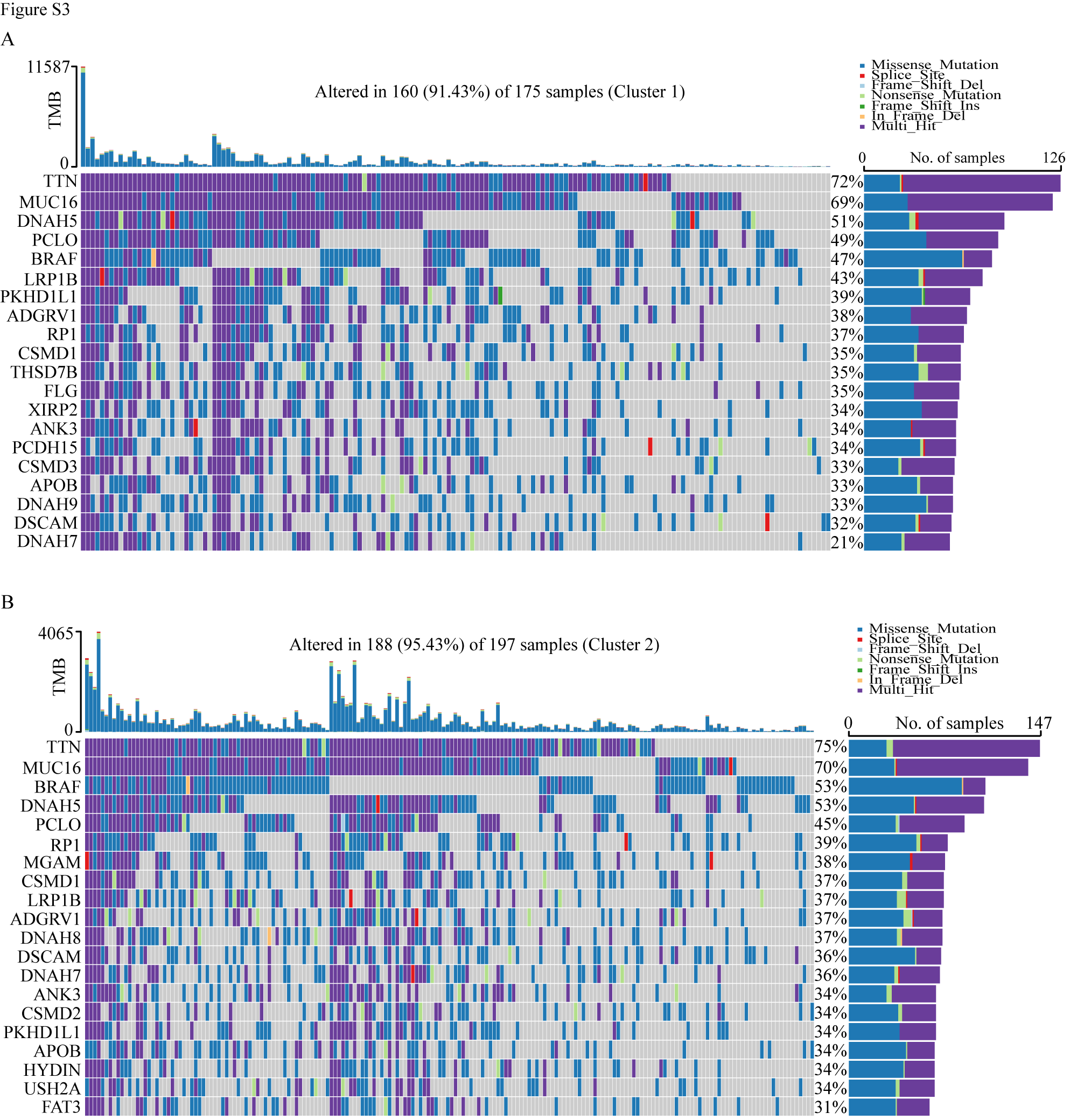

Supplement: Supplementary Figure 3 — mutation analysis of SKCM subtypes. (A) The waterfall plot showing the top 20 mutated gene of Cluster1. (B) The waterfall plot showing the top 20 mutated gene of Cluster2. Each column represents a single patient. The upper barplot showed the total tumor mutation burden (TMB), The number on the right shows the mutation frequency of each gene. The bar graph on the right shows the proportion of each mutation type. [file Image_3.tif]

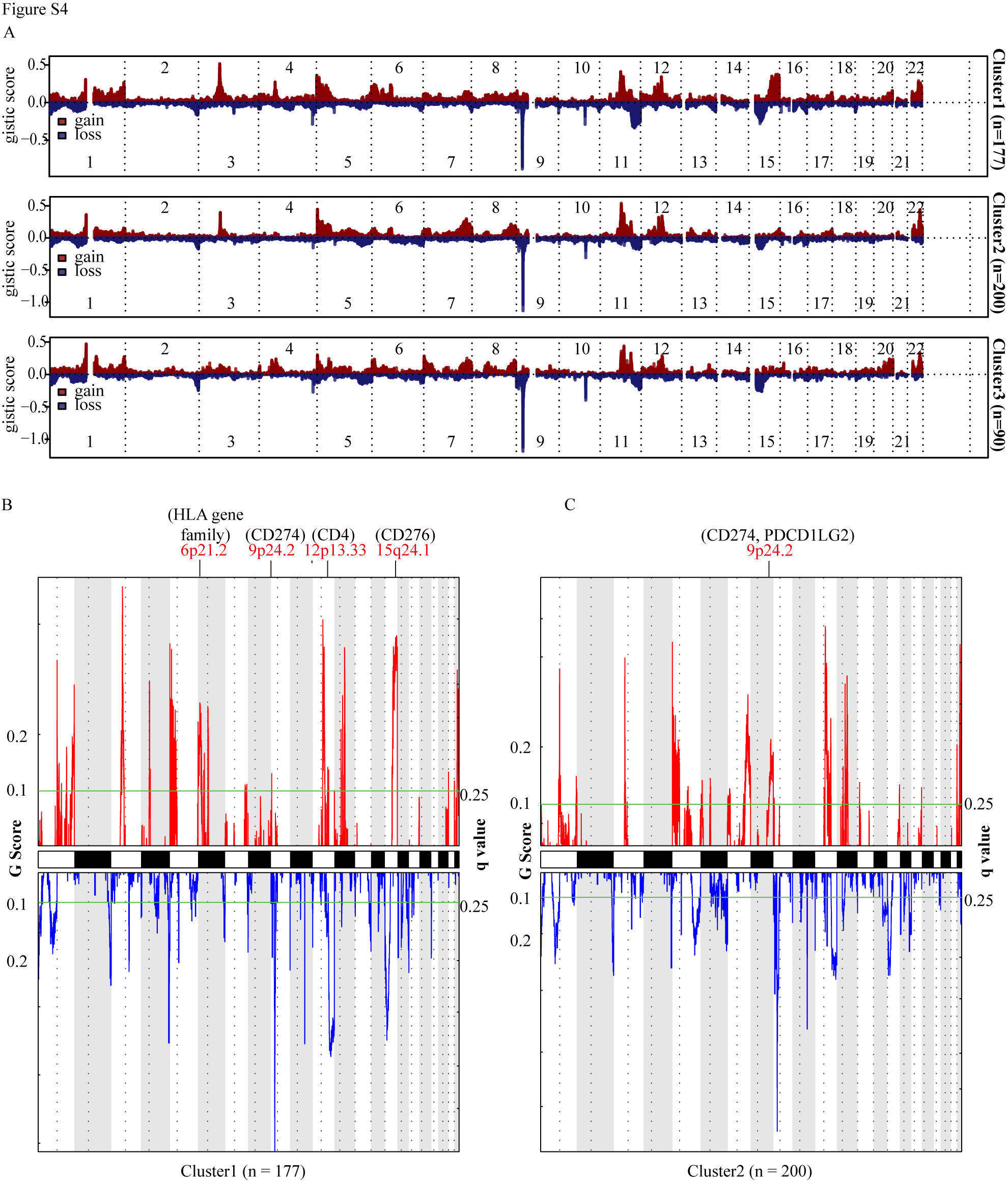

Supplement: Supplementary Figure 4 — Copy number variation (CNV) analysis of SKCM subtypes. (A) The differences in gistic score among the three subtypes. Copy number amplification or deletion is shown in red or blue respectively. (B) GISTIC 2.0 analysis showing the amplifications and deletions in Cluster1. (C) GISTIC 2.0 analysis showing the amplifications and deletions in Cluster2. Chromosomal locations of peaks of significantly gains (red) and losses (blue) are shown. The q-value, which indicates statistical significance, is displayed at the bottom of graph. Areas with q-values< 0.25 (green lines) are considered significantly altered. The locations of the peak regions of highest copy number change and the known immune checkpoint genes within these peaks are indicated. [file Image_4.tif]

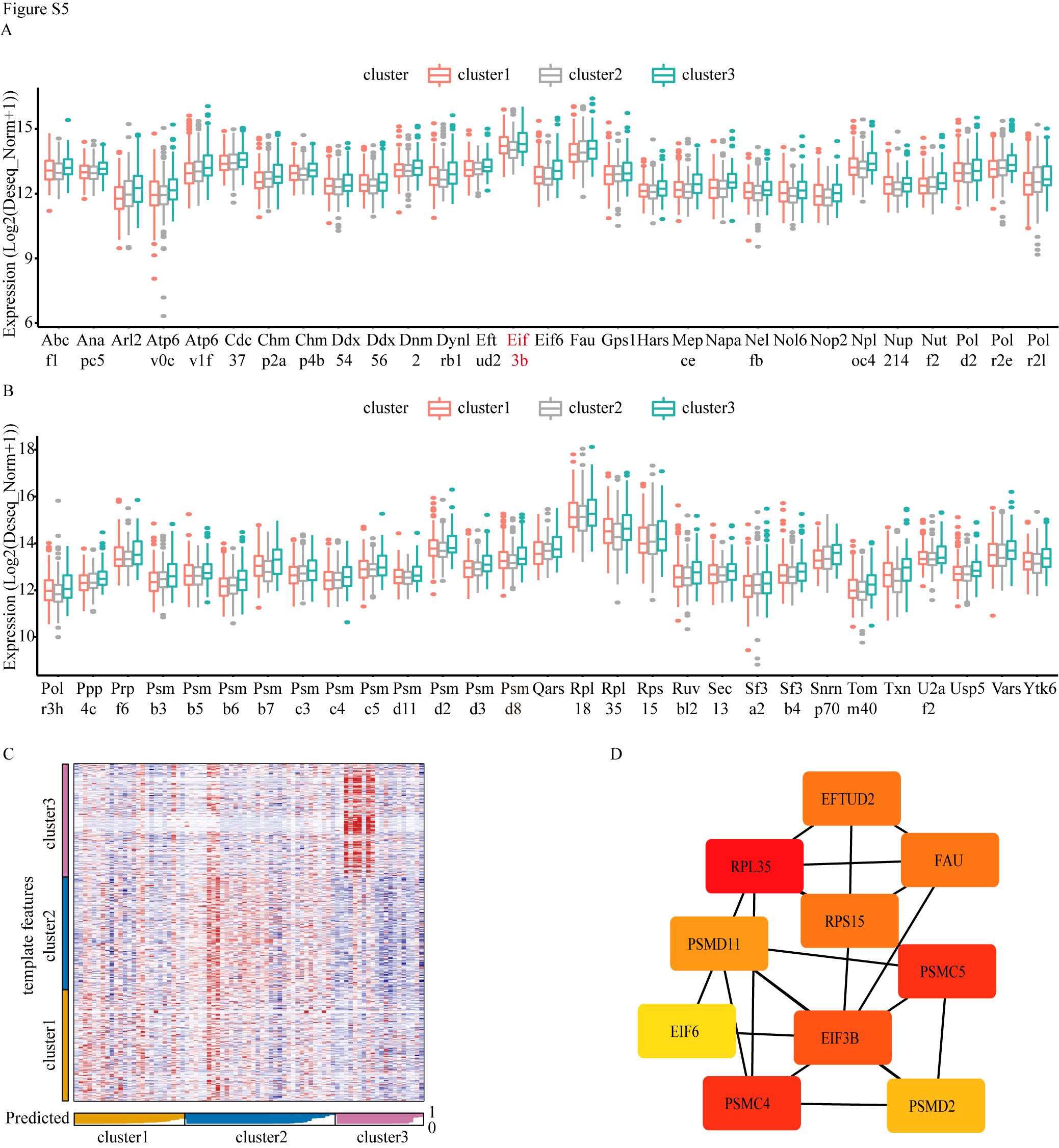

Supplement: Supplementary Figure 5 — EIF3B as a hub gene of Cluster3 specific gene. (A, B) Boxplots showing that expression of 60 intersected genes (Cluster3 specific upregulated genes and essential genes in the DepMap database) across the three subtypes. (C) Predicted classification of merged GEO (GSE91060+78220) anti-PD-1 immunotherapy dataset was performed using the TCGA-derived cluster specific upregulated genes and Nearest Template Prediction (NTP) algorithm. (D) The networks of hub genes in the 60 intersected genes (Cluster3 specific upregulated genes and essential genes in the DepMap database) were identified using the Radiality method. [file Image_5.tif]

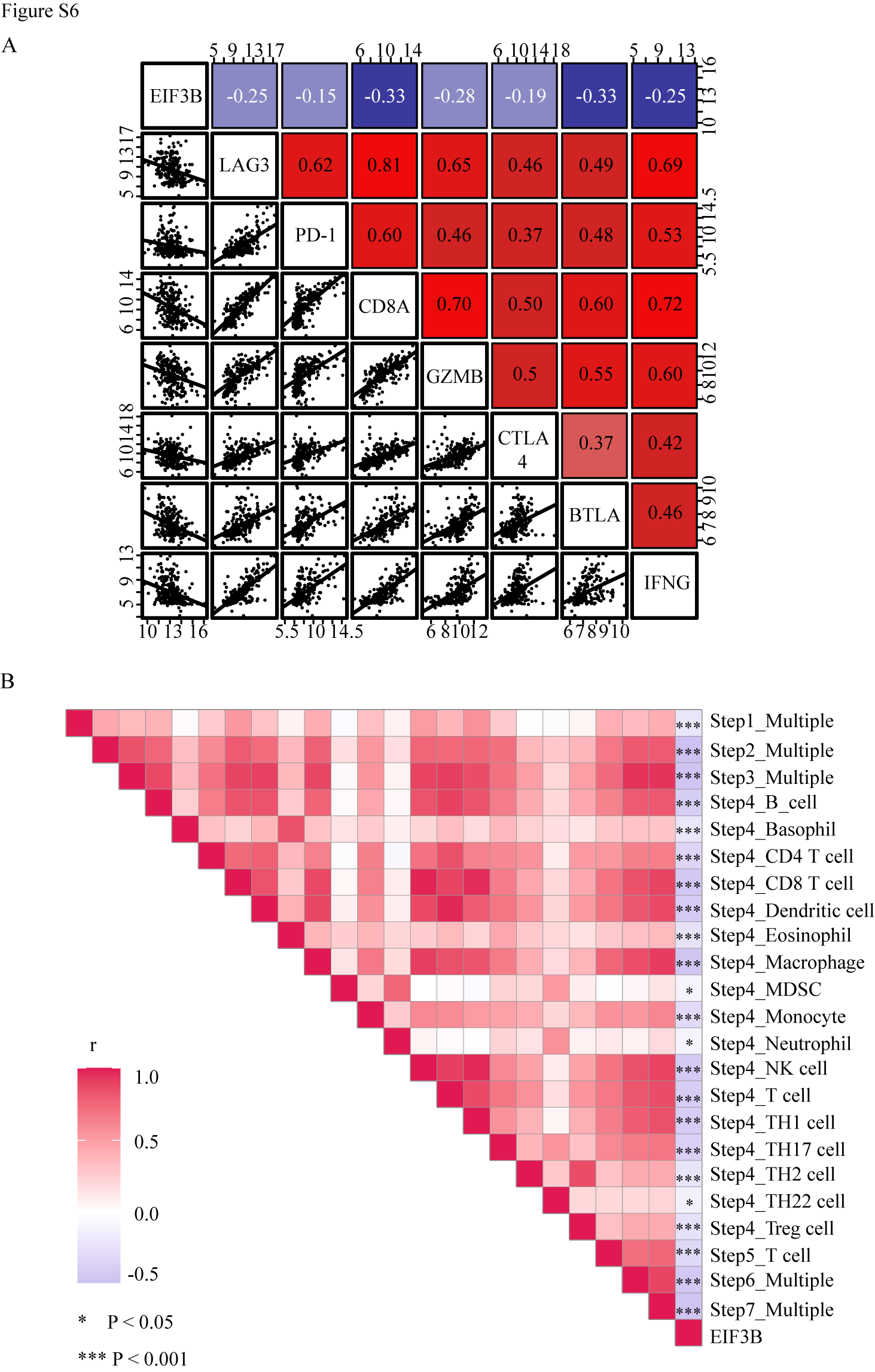

Supplement: Supplementary Figure 6 — Roles of EIF3B in predicting immune response. (A) Correlations among EIF3B, LAG3, PD-1, CD8A, GZMB CTLA4, BTLA and IFNG levels in merged GEO dataset. (B) Correlations between EIF3B and the steps of the cancer immunity cycle in TCGA SKCM cohort. The Pearson correlation test was used to calculate the correlation coefficients. [file Image_6.tif]

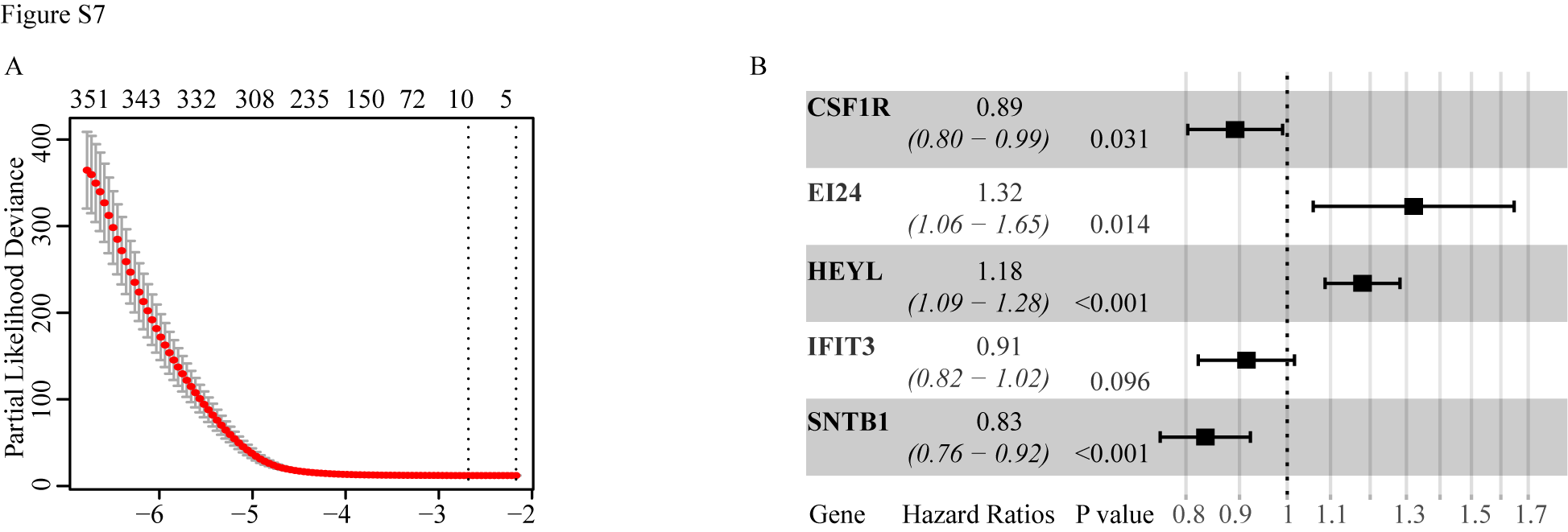

Supplement: Supplementary Figure 7 — (A) 10-fold cross-validation to select tuning parameters for LASSO models. (B) Forest plot of hazard ratios from multivariable Cox proportional hazard regression model. [file Image_7.tif]

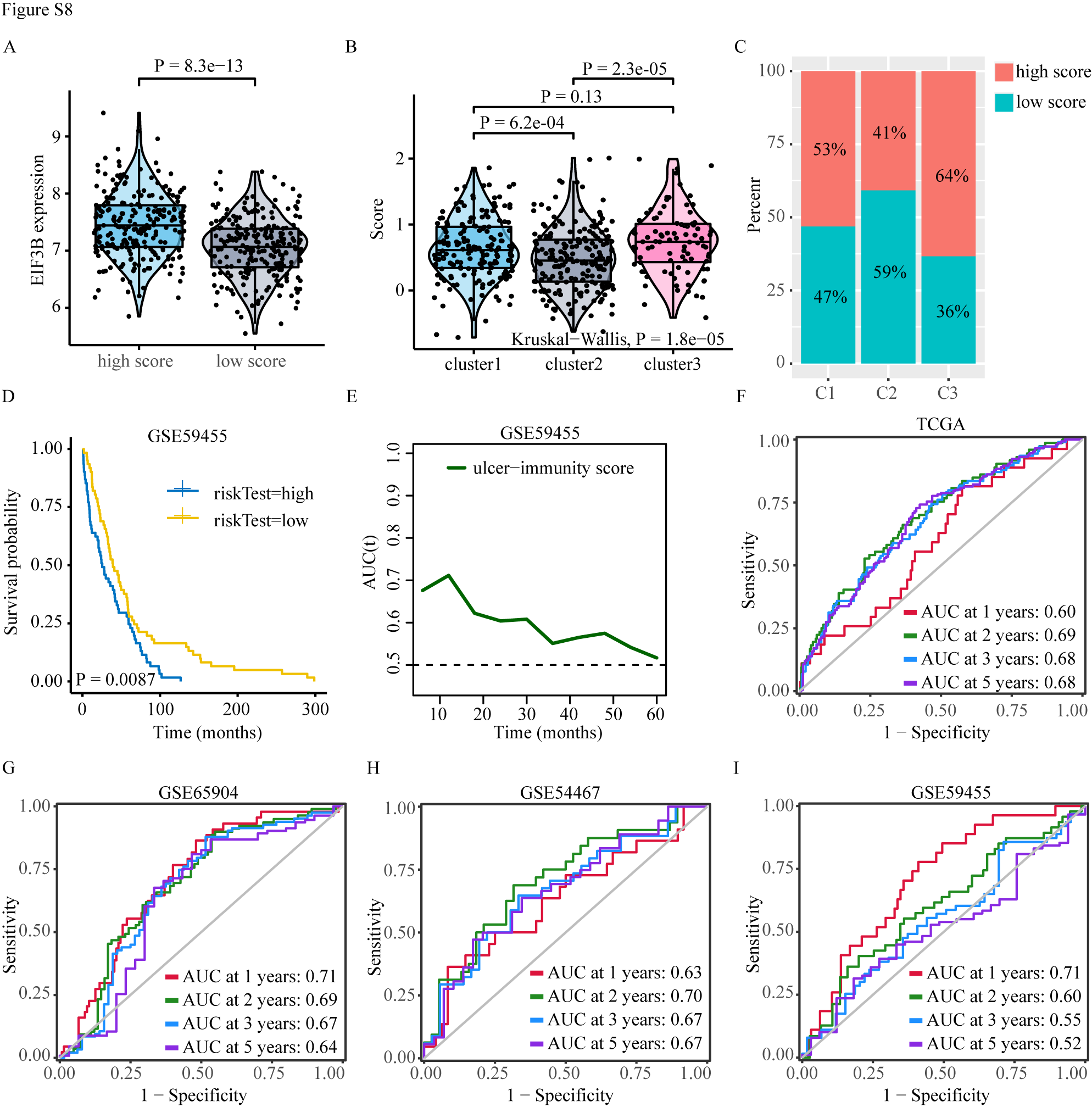

Supplement: Supplementary Figure 8 — (A) The differences of EIF3B expression between high-risk and low-risk groups in TCGA dataset, the Student-t test was used to calculate the statistical differences. (B) The differences of risk score between different subtypes in TCGA dataset, the Kruskal-Wallis test was used to calculate the statistical differences. (C) The percentage of patients with different risk score in different cluster. (D) Kaplan–Meier analysis showing the association between risk score and SKCM patient overall survival (OS) in GSE59455. (E) Time-dependent AUC value in GSE59455. (F–I) AUC values for 1-, 2-, 3- and 5-year OS in TCGA and GSE65904, GSE59455, GSE54467.. [file Image_8.tif]
