# Supplementary material for: Establishing a Prognostic Model Based on Ulceration and Immune Related Genes in Melanoma Patients and Identification of EIF3B as a Therapeutic Target
Source: Front Immunol. 2022 Feb 22;13:824946. doi: 10.3389/fimmu.2022.824946 (PMC8901887; doi:10.3389/fimmu.2022.824946)
Supplement: Supplementary file 9 [file Table_1.docx]

Table S1 Clinicopathological characteristics in TCGA SKCM cohort and GEO validation cohort

| characteristics | Freq |
| --- | --- |
| **TCGA SKCM (n=467)** |  |
| Gender, No.(%) |  |
| FEMALE | 178 (38%) |
| MALE | 289 (62%) |
| Age, No.(%) |  |
| ≥ 65 | 170 (36%) |
| < 65 | 289 (62%) |
| Unknown | 8 (2%) |
| BMI, No.(%) |  |
| ≥ 30 | 78 (17%) |
| < 30 | 170 (36%) |
| Unknown | 219 (47%) |
| Ulceration, No.(%) |  |
| Yes | 174 (37%) |
| No | 116 (25%) |
| Unknown | 177 (38%) |
| radiation_therapy, No.(%) |  |
| YES | 49 (11%) |
| NO | 420 (89%) |
| clark_level, No.(%) |  |
| I-III | 99 (21%) |
| IV-V | 220 (47%) |
| Unknown | 148 (32%) |
| breslow_depth, No.(%) |  |
| ≥ 2.0 cm | 229 (49%) |
| < 2.0 cm | 129 (27%) |
| Unknown | 109 (24%) |
| pathologic_T, No.(%) |  |
| T3-4 | 242 (52%) |
| T1-2 | 118 (25%) |
| Unknown | 107 (23%) |
| pathologic_N, No.(%) |  |
| N1-3 | 177 (38%) |
| N0 | 233 (49%) |
| Unknown | 57 (13%) |
| pathologic_M, No.(%) |  |
| M1 | 25 (5%) |
| M0 | 414 (89%) |
| Unknown | 28 (6%) |
| **GSE65904+19234+59455 (n=398)** |  |
| Gender, No.(%) |  |
| FEMALE | 150 (39%) |
| MALE | 248 (61%) |
| Age, No.(%) |  |
| ≥ 65 | 198 (49%) |
| < 65 | 179 (45%) |
| Unknown | 21 (6%) |
