# Supplementary material for: Establishing a Prognostic Model Based on Ulceration and Immune Related Genes in Melanoma Patients and Identification of EIF3B as a Therapeutic Target
Source: Front Immunol. 2022 Feb 22;13:824946. doi: 10.3389/fimmu.2022.824946 (PMC8901887; doi:10.3389/fimmu.2022.824946)
Supplement: Supplementary file 10 [file Table_2.docx]

Table S2 Univariate and multivariate analyses of clinicopathological characteristics, Cluster, and EIF3B with overall survival in TCGA SKCM cohort and GEO cohort

|  | Univariate analysis |  | Multivariate analysis |  |
| --- | --- | --- | --- | --- |
|  | HR (95% CI) | P value | HR (95% CI) | P value |
| **TCGA SKCM (n=467)** |  |  |  |  |
| Gender  (Female VS Male) | 1.106(0.906-1.349) | 0.322 |  |  |
| Clark_level  (IV-V VS I-III) | 1.682(1.313-2.154) | <0.001 | 1.383(1.053-1.816) | 0.02 |
| Pathologic_stage  (III-IV VS I-II) | 1.634(1.299-2.055) | <0.001 | 1.048(0.648-1.695) | 0.847 |
| BMI  (≥ 30 VS < 30) | 0.936(0.682-1.284) | 0.681 |  |  |
| Age  (≥ 65 VS < 65) | 0.951(0.78-1.158) | 0.616 |  |  |
| Radiation_therapy  (Yes VS < No) | 1.253(0.949-1.653) | 0.111 |  |  |
| Breslow_depth  (≥ 2.0 VS < 2.0) | 0.932(0.749-1.161) | 0.531 |  |  |
| Cluster3 | 2.111(1.632-2.73) | <0.001 | 2.09(1.549-2.82) | <0.001 |
| EIF3B | 1.567(1.235-1.988) | <0.001 | 1.379(1.019-1.866) | 0.037 |
| **GSE65904+19234+59455(n=399)** |  |  |  |  |
| Gender  (Female VS Male) | 1.225(0.974-1.541) | 0.082 |  |  |
| Age  (≥ 65 VS < 65) | 1.152(0.928-1.43) | 0.201 |  |  |
| Cluster3 | 1.843(1.252-2.714) | 0.002 | 1.741(1.176-2.577) | 0.006 |
| EIF3B | 1.459(1.157-1.841) | 0.001 | 1.402(1.109-1.772) | 0.005 |
